# Differences in the Predictors of Reading Comprehension in First Graders from Low Socio-Economic Status Families with Either Good or Poor Decoding Skills

**DOI:** 10.1371/journal.pone.0119581

**Published:** 2015-03-20

**Authors:** Edouard Gentaz, Liliane Sprenger-Charolles, Anne Theurel

**Affiliations:** 1 Laboratoire de Psychologie et Neurocognition (CNRS) and Univ. Grenoble Alpes, Grenoble, France; 2 Faculté de Psychologie et des Sciences de l’Education, Université de Genève, Geneva, Switzerland; LPNC, Centre national de la recherche scientifique, Grenoble, France; 3 Laboratoire de Psychologie Cognitive (CNRS), Aix-Marseille Université, Marseille, France; University of Leicester, UNITED KINGDOM

## Abstract

Based on the assumption that good decoding skills constitute a bootstrapping mechanism for reading comprehension, the present study investigated the relative contribution of the former skill to the latter compared to that of three other predictors of reading comprehension (listening comprehension, vocabulary and phonemic awareness) in 392 French-speaking first graders from low SES families. This large sample was split into three groups according to their level of decoding skills assessed by pseudoword reading. Using a cutoff of 1 SD above or below the mean of the entire population, there were 63 good decoders, 267 average decoders and 62 poor decoders. 58% of the variance in reading comprehension was explained by our four predictors, with decoding skills proving to be the best predictor (12.1%, 7.3% for listening comprehension, 4.6% for vocabulary and 3.3% for phonemic awareness). Interaction between group versus decoding skills, listening comprehension and phonemic awareness accounted for significant additional variance (3.6%, 1.1% and 1.0%, respectively). The effects on reading comprehension of decoding skills and phonemic awareness were higher in poor and average decoders than in good decoders whereas listening comprehension accounted for more variance in good and average decoders than in poor decoders. Furthermore, the percentage of children with impaired reading comprehension skills was higher in the group of poor decoders (55%) than in the two other groups (average decoders: 7%; good decoders: 0%) and only 6 children (1.5%) had impaired reading comprehension skills with unimpaired decoding skills, listening comprehension or vocabulary. These results challenge the outcomes of studies on “poor comprehenders” by showing that, at least in first grade, poor reading comprehension is strongly linked to the level of decoding skills.

## Introduction

The present study follows a previous one in which the relative contributions of the main predictors of reading comprehension were investigated in almost 400 French first graders from low SES families [1]. The results indicated that, at the end of Grade 1, decoding skills and listening comprehension made a significant and independent contribution to reading comprehension, the contribution of decoding skills being clearly greater than that of listening comprehension. The originality of the present study is to examine the factors that explain reading comprehension in the first graders from the previous study with good, average or poor decoding skills, these skills being assessed with items that should be read using grapheme-phoneme correspondences (GPC) that is pseudowords.

The authors of the simple view of reading (SVR), in one of their first publications [2], acknowledge that the term “decoding” can have several means: “Some equate it with sounding out, others with (context-free) word recognition. Our position is closer to the latter, for we believe that sounding out is (…) at least a primitive form of decoding (…). Yet we are reluctant to equate decoding with word recognition… But we firmly believe that word recognition skills (in an alphabetic orthography) is fundamentally dependent upon knowledge of letter-sound correspondence” (pp.6–7). In another study [3], they indicate (p. 131) that “an adequate measure of decoding skill must tap this ability to access the mental lexicon for arbitrary printed words (e.g. by assessing the ability to pronounce isolated real words). However, for beginning readers, who must acquire a phonological-based system, an adequate decoding measure must assess skill in deriving appropriate phonological-based representations of novel letter strings (e.g. by assessing the ability to pronounced isolated pseudowords).”

The other main reason for the choice of a pseudoword reading task is that, according to some researchers [4–5], good decoding skills constitute a bootstrapping mechanism for reading acquisition (including not only the reading of irregular words but also reading comprehension). This is because children who have understood well how to use GPCs to read have more cognitive resources available to process both the irregularities of these correspondences and the meaning of what they are reading. Moreover, the role of decoding skills in reading acquisition should be facilitated by the transparency of GPCs in the language in which children learn to read [6–8]. There are currently no studies that have considered this perspective to assess the predictors of reading comprehension.

### Predictors of reading comprehension based on the simple view of reading

According to the simple view of reading (SVR [2–3]), reading comprehension is the product of two skills: listening comprehension and decoding. That model is supported by empirical evidences from different studies in which decoding skills were assessed with pseudowords [1] [9–10]. Some of these studies have also shown that the relative contribution of each of the two main predictors of reading comprehension depends on children’s grade level: between early and later grades, the contribution of listening comprehension increases whereas that of decoding skills decreases. For instance, in a longitudinal study [9], only the contribution of decoding skills was significant in Grade 1, whereas both decoding skills and listening comprehension made a significant and independent contribution to reading comprehension in Grade 2, the contribution of decoding skills being greater than that of listening comprehension.

Contradictory results have been found in other studies. For instance, in a study with first and second graders [11], it was observed that, in both grades, listening comprehension and decoding skills predicted reading comprehension; however, listening comprehension was a more powerful predictor of reading comprehension than decoding skills. An even more surprising result comes from a longitudinal study in which word-level reading skills were assessed by two tasks: one using isolated words, the other using words in context [12]. Neither of these word-level reading skills significantly explained reading comprehension, even in the first wave of the longitudinal study, when the children were 7–8 years old.

At least two reasons may explain these surprising results. The first is related to the test used to assess decoding skills in studies [12] and [13]. In these studies, word-level reading skills were assessed with the Neale test [14] which provides measurements of accuracy and rate (i.e. the average number of words read per minute) for the reading of words in context. However, we now know that this type of measurement is not very sensitive for differentiating between good readers and poor readers, and more specifically between good readers and dyslexics, who use the context to compensate for their deficit in the reading of isolated words [15–16]. Moreover, as the Neale test was also used to assess reading comprehension, children’s word reading errors were corrected during their reading and words they misread were subsequently told to them. Therefore, the weight of decoding skills on reading comprehension was minimized and the link between reading and listening comprehension was strengthened.

The second reason is likely the selection of children in at least two studies using a large sample aimed to be representative of the primary school children population [12–13]. In fact, all children included in these studies had word-level reading skills that were within 18 months of their chronological age, with either extremely poor readers or extremely good readers being excluded.

To prevent this, which we consider to be a limitation, in the present study we have included the entire population (without selection) that has been examined in a former study [1]. In addition, word-level reading skills were assessed with pseudowords, items for which weaknesses in the knowledge of GPCs cannot be compensated by either contextual pieces of information or lexical knowledge.

### The case of poor comprehenders with good decoding skills

Some studies have highlighted the presence of dissociations between reading comprehension and decoding skills, showing that children can be poor comprehenders despite having good decoding skills [17–20]. However, a review of the literature on “poor comprehenders” [21] reported some studies [18–19] clearly showing that poor comprehenders with good decoding skills have quite marked difficulties on a wide range of oral language measurements, as compared to typically developing children.

This is also the conclusion of a recent study in which the relationships between reading comprehension, listening comprehension and decoding skills were examined in a large population of primary school children from low SES families [22]. In this study, among 143,671 first graders, very few had severe difficulties in reading comprehension (scores in the 5th percentile in a test assessing reading comprehension) while also having unimpaired listening comprehension and decoding skills (scores above 25th percentile in two tests: one assessing the level of vocabulary, the other pseudoword reading). This was the case for only 173 children out of the 5286 very poor comprehenders found among these first graders (0.12% of the entire population).

Some inconsistencies in the results of the studies on poor comprehenders might be due to the threshold chosen to qualify reading comprehension as being poor and word-level reading skills as being unimpaired. In some studies [e.g., 22] children with a reading comprehension deficit were those with scores at or below the 5th percentile, those characterized by unimpaired word-level reading skills having scores at, or above, the 25th percentile. In other studies [e.g., 17] poor comprehenders were defined as children who scored at least one standard deviation below the mean (i.e., below the 16th percentile) on a measurement of reading comprehension, with a same cutoff for poor word-level reading skills or listening comprehension. In some other studies [e.g., 18] poor comprehenders with unimpaired word-level reading skills were the children who scored below 25th percentile in reading comprehension and above the 40th percentile in word-level reading skills. Finally, according to another cutoff, poor versus good comprehenders were the children with scores in the lowest versus the highest third, the cutoff for poor versus good decoders being based on the median score on decoding, as in a study with 106 first graders [11]. Therefore, it is not surprising to find 35 poor comprehenders among these children, with 18 good versus 17 poor decoders.

As stated above, in the present study we have considered the entire population that was already examined in a previous study [1]. To assess the role of decoding skills on reading comprehension, this population was separated into 3 groups with a cutoff of one standard deviation to define the good and the poor decoders versus the average decoders.

### Assessments of reading and listening comprehension

A requirement of the SVR model is that the levels in reading and listening comprehension should be assessed by similar tasks [3]. This choice could also avoid a potential underestimation of the contribution of listening comprehension to reading comprehension [1] [23]. Furthermore, tests with short utterances (such as the PIAT [24]) captured more of the variance in reading comprehension than tests with longer passages (such as the GORT [25]), especially in young participants [26]. As noted by some researchers [21], this type of test is routinely used to assess oral language comprehension in children with specific language impairments. This is also the case in some studies conducted in the framework of the SVR model for the assessment of either listening comprehension (e.g., [19] [27]) or reading comprehension (e.g. [26–28]) or both (e.g. [1]).

Therefore, in the present study we used a task similar to the PIAT to assess both reading and listening comprehension. The main objective intended is to test an extended version of the SVR model which, for reasons explained below, also includes measurements of oral vocabulary and phonemic awareness.

### Vocabulary and reading acquisition

Listening comprehension, one of the two key components of the SVR model, may be considered to encompass different levels of linguistic knowledge, and thus potentially confounding other aspects of oral language involved in reading comprehension. It may be the case of vocabulary, a lexical skill which has been examined in studies assessing an extended version of the SVR model [10] [23] [27] [29–32].

These studies have produced contradictory findings. Some indicate a very early influence of vocabulary on reading comprehension. For instance, a study with English-speaking children found that early vocabulary knowledge assessed at the beginning of kindergarten predicted early reading comprehension measured at the beginning of Grade 2 [30]. An early effect of vocabulary on reading comprehension was also observed in Grades 2 or 3 in English [23] [27] [29] [32]. In contrast, other studies with English-speaking children found no contribution of vocabulary to reading comprehension in early grades (Grade 1 [10]) but a significant contribution in later grades (Grade 6 [10]). Inconsistent results were observed even among English-speaking children with poor reading comprehension skills: some studies have found their level of vocabulary to differ from that of the control children [31], while others have not [19]. In languages with a shallower orthography than English a significant effect of vocabulary on reading comprehension was observed very early: at the end of Grade 1 in French [1] or Grade 2 in Dutch [33]. It should be noted that the chronological age of French first graders was almost the same as the one of English-speaking children from grade 2 (Age 7) and that of Dutch second graders almost the same as the one of English-speaking children from grade 3 (Age 8).

The finding that vocabulary influences reading comprehension in English-speaking children in Grade 6 but not in Grade 1 [10] is surprising in the context of models that highlight the role of vocabulary in reading comprehension [32] [34]. This finding is all the more surprising given that the role of vocabulary on reading comprehension must depend on two factors: the consistency of GPCs and the child’s reading level. However, the effect of these factors is not supposed to be in the direction of some of the findings discussed in the previous paragraph: it should be greater in languages with a deep orthography (English) than in languages with a more transparent orthography (French or Dutch), especially at the beginning of reading acquisition, when children’s grasp of GPCs is still limited. For instance, if what the child produces on a first attempt at reading a word does not sound like something that is in his/her oral vocabulary, then he/she has to change one or more GPCs and try again. These operations are thought to be facilitated by a good vocabulary [27]. Therefore, the effect of vocabulary on reading comprehension should be high at the beginning of reading acquisition in languages with a deep orthography. In addition, whatever the level of opacity of the orthography, its effect should be higher in poor than in good decoders.

To clarify the role of vocabulary in reading comprehension the present study includes an assessment of that skill. We used a test in which receptive vocabulary was assessed by the choice of the picture that corresponds to a word spoken by the examiner, as in most studies conducted in the framework of the extended SVR model (e.g. [10] [27] [30] [32]).

### Phonemic awareness and reading acquisition

The classical phonological explanation of success and failure in the acquisition of reading skills is based on the role of phonemic awareness in that acquisition (for reviews see [7–8], [35]). This skill was assessed within the framework of the extended version of the SVR model in several studies ([10] [12–13] [23] [30]). Some of these studies found no evidence of a direct link between reading comprehension and phonemic awareness, whatever the age or the grade of the participants (with either young children [9] [12] [30] or older children [13] [23]). Alternatively, in a study with children schooled in Grades 1 and 6 [10] the proportion of variance explained by phonemic awareness decreased with grade (from 45.4% in Grade 1 to 17.6% in Grade 6), but it was still significant in the latter grade. This was not the case in another study [23], in which the effect of phonemic awareness on reading comprehension was significant only in the younger groups (Grades 2 and 3), but not in the older groups (Grades 6 and 7).

These inconsistent results highlight the need to clarify the role of phonemic awareness in reading comprehension. Therefore, in the present study we included an assessment of phonemic awareness as a control task, in addition to an assessment of syllabic awareness.

### Effect of children’s SES on reading acquisition

Regarding the role of the children’s SES, delays in reading and pre-reading skills have often been reported in children from low SES families [36–37] and linguistic abilities are strongly correlated with SES [38]. Scores of low SES children, even those in the typical readers group [39–41] are low, both for spoken and written languages.

Despite the large body of evidence confirming the significant role of SES factors on reading achievement, there are very few studies on reading comprehension in children from low SES families, especially in languages with a shallower orthography than English¸ such as French [7] [42–44]. In a recent study [45], the predictors of reading comprehension were examined in a cohort of 181 French second graders from low SES families. For these children, the strongest predictors of that skill were IQ, vocabulary and attention. Unfortunately, the authors have not taken into account decoding level as a predictor of reading comprehension. It is therefore crucial to assess the relative importance of decoding skills, listening comprehension, and vocabulary on reading comprehension in French-speaking children from low SES families. This was done in the present study.

### The present study

The aim of the present study was to examine the contributions to reading comprehension of four predictors: decoding skills (measured by the reading of pseudowords), listening comprehension (assessed by a task that was similar to the one used to assess reading comprehension), vocabulary knowledge, and phonemic awareness. The participants were French-speaking children schooled in Grade 1 who were either good or poor decoders (pseudoword reading scores being one standard deviation above or below the mean of the group), or average decoders. Three main hypotheses were evaluated. Firstly, the percentages of children with impaired reading comprehension skills should be higher in poor decoders than in average decoders and very low in good decoders. Secondly, the skills expected to differentiate good and poor decoders from average decoders should be those related to decoding skills (in addition to pseudoword reading, isolated-word reading, phonemic awareness, and reading comprehension). Thirdly, the predictors of reading comprehension should not be the same in the different groups. Especially, the effect on reading comprehension of decoding skills should be higher than that of listening comprehension in poor decoders (Hypothesis 3a), the opposite being expected for good decoders (Hypothesis 3b). In addition, if the level of vocabulary plays a crucial role in the beginnings of learning to read, when children's knowledge of GPCs is still limited [27], the effect of vocabulary should be higher in poor than in good decoders (Hypothesis 3c). Finally, as the effect of phonemic awareness decreases with grade [10] [23], it would also be lower in good than in poor decoders (Hypothesis 3d).

## Methods

### Ethics statement

The present study was conducted in accordance with the Declaration of Helsinki, with the written consent of each child’s parent and it was approved by the local ethics committee of the LPNC (Laboratory of Psychology and Neurocognition, CNRS, University of Grenoble, France) and in accordance with the ethics convention between the academic organization (LPNC-CNRS) and educational organizations of France.

### Participants

392 French children (211 girls and 181 boys) with a mean age of 6 years 3 months (5 years 10 months to 6 years 9 month at the start of the school year), took part in this study. These children attended 30 different elementary school classes. They were all schooled in a “Priority Education Area”. This population corresponds to the one tested in a previous study [1]. Nonverbal IQ scores of these children, assessed using the Progressive Matrices Standard [46], were within the standards (Table 1).

**Table 1:** Summary of descriptive statistics for each of the measures.

Tests (end G1 except Nonverbal IQ)	All children		Subgroups (N) classified by level of decoding skills			ANOVAs with the 3 subgroups (Age as covariable) F(2,388)	Bonferroni *Average vs*. *Good* and Cohen’s D	Bonferroni *Average vs*. *Low* and Cohen’s D
	Mean range	Norma-tive data	Good decoders	Average decoders	Poor decoders			
	(SD) Range		N = 63	N = 267	N = 62			
Fluency: Pseudoword reading (PW-Min)	28.1		45.9	27.9	10.8	F = 426.6	*p<*.*01*	*p<*.*01*
	(11.9)		(7.9)	(6.7)	(4.2)	*p<*.*01*	D = +2.45	D = -3.05
	0–90							
Fluency: word reading (Word-Min)	39.6		66.7	38.0	18.9	F = 262.2	*p<*.*01*	*p<*.*01*
	(17.9)		(15.8)	(11.4)	(6.7)	*p<*.*01*	D = +2.07	D = -2.03
	0–128							
Reading Comprehension (% Correct)	68.9	82.2^48^	82.2	71.5	44.6	F = 83.8	*p<*.*01*	*p<*.*01*
	(20.2)		(11.9)	(16.2)	(22.6)	*p<*.*01*	D = +0.76	D = -1.37
	0–100							
Listening Comprehension (% Correct)	85.5	87.7^48^	90.0	85.6	80.2	F = 7.7	*p<*.*05*	*p<*.*01*
	(13.3)		(10.3)	(12.7)	(16.5)	*p<*.*01*	D = +0.38	D = -0.37
	28–100							
Phonemic awareness (% Correct)	61.6	73.1^50^	81.3	63.1	35.2	F = 59.0	*p<*.*01*	*p<*.*01*
	(26.5)	(18.8)	(17.5)	(23.7)	(25.1)	*p<*.*01*	D = +0.87	D = -1.14
	0–100							
Syllabic awareness (% Correct)	62.8	68.6^50^	79.7	63.9	40.5	F = 17.7	*p<*.*01*	*p<*.*01*
	(36.7)	(31.8)	(31.0)	(35.8)	(35.7)	*p<*.*01*	D = +0.45	D = -0.66
	0–100							
Vocabulary (correct responses /60)	40.2	53.6^49^	42.1	40.3	37.7	F = 4.9	p = .22	*p<*.*05*
	(7.4)	(4.7)	(7.6)	(7.4)	(7.0)	*p<*.*01*	D = +0.25	D = -0.36
	16–56							
Nonverbal IQ (correct responses /36)	20.8	21.0^46^	21.6	21.0	19.0	F = 5.4	p = .89	*p<*.*01*
	(4.6)	(5.0)	(4.8)	(4.6)	(4.0)	*p<*.*01*	D = +0.14	D = -0.47
	14–34							

Because our goal was to examine the role of decoding skills on reading comprehension, the children were separated into subgroups according to their level of decoding skills. This level was assessed with pseudowords; items for which weaknesses in the knowledge of GPCs cannot be compensated by lexical knowledge (see the following section for a description of the task). In addition, we decided to kept the entire population (and not only the average children, as in [12–13], for instance). We assumed that this choice would have better ecological validity for this type of study. Finally, we used a conventional cutoff of 1 SD above or below the mean of the entire group to define the groups of good and poor decoders. With this cutoff, 63 good decoders, 62 poor decoders, and 267 average decoders were identified. Their mean scores (and SDs) for decoding skills were respectively: 45.88 (7.90), 27.91 (6.72), and 10.87 (4.17). As explained in a previous article [47] our sample is representative of the French population schooled in “a priority education area”. The representativeness of our sample was examined by the comparisons of the results obtained in mathematics and French in the 2011 national assessments by the schools in which were enrolled the students of the present study compared to a larger national sample of same schools.

### Procedure and assessments

Each child was administered tasks assessing nonverbal IQ, vocabulary, listening and reading comprehension, isolated word and pseudoword reading, phonemic and syllabic awareness. All tasks were administered by psychologists who were trained and supervised, and were administered to each child individually, except for nonverbal IQ, which was assessed in groups of 4–6 children, depending on the size of class. Assessments took place at the end of Grade 1 (except for nonverbal IQ assessed at the beginning of that grade), in a quiet room in the schools, and lasted approximately 2 x 30 minutes (with a break of about 40 min) per child. All tasks were administered on the same day for all children of the same class. The task order was established so as to best maintain the attention and the interest of the children. First we assessed listening comprehension, pseudowords and word reading, and vocabulary. After the break, phonological awareness and reading comprehension were evaluated.

#### Fluency in isolated word and pseudoword reading

Fluency is a composite measurement which takes into account accuracy and time: very often (as in the present study) the number of items correctly read in one minute. There were 60 pseudowords made-up of 1 to 7 letters and 60 high frequency words [51], including 10 irregular words. The words were matched to the pseudowords in length and orthographic difficulty. Interestingly, the correlation between the word reading task and pseudoword reading task was high (.91).

#### Reading comprehension

Reading comprehension was assessed with a task containing 14 short utterances (length between 5 to 9 words) extracted from the ECoSSe test: Epreuve de Compréhension Syntactico-Sémantique (Test of Syntactic and Semantic Comprehension [48]). Six structures were used with at least two utterances for each structure: two active and two passive sentences, two utterances for which the choice of the good picture requires the processing of a pronoun, two utterances with two negations; four utterances with a spatial term; and two utterances with a relative clause and a spatial term. For examples: Passive sentences (Le garcon est poursuivi par le mouton [The boy is chased by the sheep]); Utterances with a pronoun (La vache les regarde [The cow watches them]); Utterances with two negations (Le garcon n’a ni chapeau ni chaussure [The boy has not hat and no shoes]); Simple and complex utterances with a spatial term (Le boîte est derrière la tasse [The box is behind the cup]; L’étoile qui est dans le cercle est rouge [The star which is in the cercle is red]. The child had to read each utterance aloud. He/she was then shown a page with four pictures and had to choose the picture which matched the utterance he/she had read, without the possibility of re-reading it. The reading comprehension scale had 14 points. Percentages of correct responses were calculated.

#### Listening comprehension

The task used to assess listening comprehension was composed of 14 utterances from the ECoSSe test [48]. These utterances were similar to those used in the reading task, but with a different wording (for instance, “the box is behind the cup” and “the pencil is in front of the box” instead of “the cup is in front of the box” and “the pencil is behind the box”). The procedure was the same as the one used in the reading task, except that the examiner first presented each utterance orally; then the child was shown a page with four pictures, his/her task being to select the picture which matched the utterance he/she had heard, without the possibility of hearing it again. Percentages of correct responses were calculated.

#### Vocabulary

The level of vocabulary was assessed by a standardized receptive vocabulary test [49], in which the children had to choose the picture (out of six) that matched the word read by the examiner. The test consisted of 30 items, each scored on a scale from 0 to 2 (2 points were awarded for the choice of the correct response and 1 point for the approximate response). The maximum score was therefore 60.

#### Phonemic awareness

The task, as the control task at the syllabic level, is from EVALEC [50]. The child had to delete the first element of a pseudoword, either the first syllable of 10 trisyllabic pseudoword with a consonant-vowel structure (CV) or the first phoneme of a one-syllable pseudoword with 3 phonemes (12 CVC and 12 CCV). We used a phonemic deletion task because, as explained in a recent meta-analysis [35, p.327], many studies used that type of task to assess phonemic awareness, and it was established that this is a measure with high reliability. Percentages of correct responses were calculated for each of these two tasks.

## Results

Firstly, we examined the differences (ANOVAs, *t*-test and Cohen’s *D*) between the three groups defined according to their level of decoding (good, average and low) on reading comprehension and its predictors: in addition to decoding skills (fluency in pseudoword reading), listening comprehension, vocabulary, phonemic awareness (with a control task of syllabic awareness) and nonverbal IQ. Then, we examined the prevalence of reading comprehension deficits in the same three groups. Finally, we looked at the predictors of reading comprehension among these groups (correlation and regression analyses).

### Characteristics of the population and group differences

Table 1 presents the results for the entire population. Our sample was comparable to standards for nonverbal IQ: *t*(391) = -0.9; *p* = .36. However, for the five tasks involving linguistic knowledge for which standards were available, the means in our sample were lower than the standards: all *p*s were equal or above. 01 (for vocabulary, listening and reading comprehension, phonemic and syllabic awareness, respectively, *t*(391): -35.7, -3.3, -13, -181.3 and -336.2). These results confirmed that the low SES children in this study showed impairments in both their spoken and written language as observed in previous studies on English-speaking [37] or French-speaking [52] children. Otherwise, our sample was comparable to the standards for nonverbal IQ as observed in children from low SES families in previous studies [36].

Among these children, 267 were classified as average decoders, 63 as good readers and 62 as poor readers. ANOVAs were conducted with these three groups as the between-subjects factor and age as a co-variable. When the effect of group was significant, t-tests were computed to examine the differences between the poor or the good decoders versus the average decoders; significance after Bonferroni correction was used, and the size of the differences was examined with Cohen’s *d*.

Table 1 also presents the descriptive statistics for the three groups, and the outcomes of the ANOVAs and t-tests. The main group effects were always significant (i.e., for fluency in pseudoword and word reading, reading and listening comprehension, phonemic and syllabic awareness, vocabulary and nonverbal IQ, Fs(2,388) = 426.6, 262.21, 83.8, 7.7, 59.01, 17.74, 4.9 and 5.4, all ps < 0.01. T-tests (with Bonferroni corrections) indicated non-significant group differences only for nonverbal IQ and vocabulary between good versus average decoders. However, consistently with our expectations, the strongest differences between average versus good or poor decoders were observed in the skills supposed to be linked to decoding skills (word reading, reading comprehension and phonemic awareness) and, of course, in decoding skills. In these eight cases, Cohen's ds were large: more than 2 for decoding skills and word reading (average versus good decoders: +2.45 and +2.07; average versus poor decoders: -3.05 and -2.03); almost 0.8 for reading comprehension (respectively, +0.76 and -1.37) and for phonemic awareness (respectively, +0.87 and -1.14). Among the eight other differences between the groups, five were either non-significant (between average versus good decoders for nonverbal IQ and for vocabulary) or small (average versus good and poor decoders for listening comprehension: +0.38 and -0.37; average versus poor decoders for vocabulary: -0.36). The three other differences were of a medium size: the difference between average versus good and poor decoders for syllabic awareness (+0.45 and -0.66), and the difference between average and poor decoders for nonverbal IQ (-0.47).

### Prevalence of deficits in reading comprehension in the three groups

The prevalence of the deficits was established in relation to the scores of the entire population (with 392 children). A child was considered to suffer from a deficit in a specific domain when his/her scores in that domain were one standard deviation below the mean of the group. Among children identified as having a reading comprehension deficit, we looked at those with a deficit in pseudoword reading (by definition, 100% in the group of poor decoders, and 0% in the other two groups) together with a deficit in one of the related skills (listening comprehension or vocabulary), or in both listening comprehension and vocabulary.

There were 34 children (54.8%) with poor reading comprehension skills in the group of poor decoders, only 18 children (6.7%) in the group of average decoders, and none in the group of good decoders. Among the 34 poor decoders with poor reading comprehension, 20 (58.8%) had deficits in either listening comprehension or vocabulary (11 children with a deficit in listening comprehension, 4 with a deficit in vocabulary, and 5 with a deficit in both listening comprehension and vocabulary). Among the 18 average decoders with poor reading comprehension, 11 (61.1%) has deficits in either listening comprehension or vocabulary (5 children with a deficit in listening comprehension, 3 with a deficit in vocabulary, and 3 with a deficit in both listening comprehension and vocabulary). The proportion of children with impaired scores in reading comprehension despite having unimpaired skills in decoding, listening comprehension and vocabulary was very small: 6 children (1.5% of the entire population).

### Correlation and regression analyses

To perform the analyses of correlations, observed scores for the variables of interest were standardized (M = 0; SD = 1). We have considered the scores in reading comprehension and its main predictors: listening comprehension, vocabulary, decoding skills (assessed with pseudoword reading), and phonemic awareness (assessed with a control task on syllabic awareness), nonverbal IQ also being taken into account. Table 2 shows the correlations between the variables of interest and the performance on the reading comprehension task. All but six correlations were significant: four in the poor decoder group (.22 and. 24 for listening comprehension and vocabulary;. 24 for syllabic awareness;. 09 for nonverbal IQ) and two in the good decoder group (.25 for decoding skills and. 28 for phonemic awareness).

**Table 2:** Correlation matrices between reading comprehension and the other variables in the three groups.

	Poor decoders	Average decoders	Good decoders
1-Reading comprehension			
2-Nonverbal IQ	0.09	**0.20** ^*^	**0.36** ^*^
3-Vocabulary	0.24	**0.41** ^*^	**0.40** ^*^
4-Listening Comprehension	0.22	**0.44** ^*^	**0.47** ^*^
5-Decoding skills (Fluency)	**0.49** ^*^	**0.40** ^*^	0.25
6-Phonemic awareness	**0.42** ^*^ ^*^	**0.40** ^*^	0.28
7-Syllabic awareness	0.24	**0.35** ^*^	0.43*

* p <. 01 after Bonferroni correction

Regression analyses were performed in order to find out which variable (inputted in the model) predicted reading comprehension in a unique and significant way. The variables considered were our four predictors of reading comprehension (in addition to decoding skills, listening comprehension, vocabulary, and phonemic awareness) and two variables that were taken into account as control variables (nonverbal IQ and syllabic awareness). In order to determine if these variables differentially predicted reading comprehension as a function of group level (poor, average, good decoders), we entered an interaction term between these variables and group level. To do so, we recoded our levels (poor, average, good decoders) in contrast codes and we created the interaction term by multiplying this new variable by our predictors.

The analyses reported in Table 3 indicated that the variables inputted in the model accounted for 58% of the variance in reading comprehension. Decoding skills was the best predictor (12.10%), followed by listening comprehension (7.28%), vocabulary (4.57%) and phonemic awareness (3.34%), nonverbal IQ and syllabic awareness adding no significant part of the variance in reading comprehension. Interaction between group level versus decoding skills, listening comprehension and phonemic awareness accounted for significant additional variance (respectively 3.56%, 1.05% and 0.99).

**Table 3:** Standard multiple regression analyses with the reading comprehension task as dependent variable and variables of interest.

R^2^ = .58	Predictor variables	*Β*	Proportion of unique variance accounted by	*P*
	Nonverbal IQ	.01	.01	.84
	Vocabulary	.16	4.57	*<*.*001*
	Listening Comprehension	.22	7.28	*<*.*001*
	Decoding	.46	12.10	*<*.*001*
	Phonemic awareness	.17	3.34	*<*.*001*
	Syllabic awareness	.07	0.74	.09
	Level	.04	0.11	.52
	Level *Nonverbal IQ	-.02	0.10	.53
	Level *Vocabulary	.05	0.49	.17
	Level *Listening Comprehension	-.08	1.05	*<*.*05*
	Level *Decoding	.18	3.56	*<*.*01*
	Level *Phonemic awareness	.11	0.99	= .05
	Level*Syllabic awareness	-.07	0.60	.13

The amount of variance on reading comprehension accounted for in each group (poor, average, good decoders) by the predictors was not the same in the different groups. Especially, the effect of both decoding skills and phonemic awareness were significant in the groups of poor and average decoders (respectively for decoding skills: 10.81% and 7.71%; for phonemic awareness: 2.67% and 2.23%) whereas in the group of good decoders the effect of decoding skills was low (1.63%) and that of phonemic awareness null (0.00%). Alternatively, listening comprehension accounted for a significant part of the variance in reading comprehension only in good and average decoders (respectively, 5.50% and 1.76%), not in poor decoders (0.21). In addition, although the interaction between group level and vocabulary was not significant, vocabulary made a significant contribution to reading comprehension only in the groups of poor and average decoders (respectively, 1.60% and 3.15%; 0.14 in good decoders).

## Discussion

### Main results in relation to the hypotheses

Three main set of results can be highlighted. The first set concerns the differences, between the groups defined by their levels of decoding skills, in reading comprehension and its predictors. In comparison with average decoders, poor decoders attained the lowest levels in all the skills assessed, whereas good decoders attained the highest (with the exception of nonverbal IQ and vocabulary). However, consistently with our second hypothesis, the strongest differences between good or poor decoders versus average decoders were observed in the tasks tapping onto skills assumed to be linked to decoding abilities. For these skills, all Cohen's *d*s were very large (+/- 2.0 for fluency in word reading) or large (almost +/-0.8 for phonemic awareness and reading comprehension). For all other tasks (vocabulary, listening comprehension, syllabic awareness and nonverbal IQ), the sizes of the effects were small (less than 0.5), with one exception (d = 0.66 in the comparison between poor and average decoders for syllabic awareness).

Secondly, in agreement with our first hypothesis, the percentages of children with impaired reading comprehension skills strongly depend on the level of decoding skills: it was higher in poor decoders (55%) than in the two other groups (average decoders: 7%; good decoders: 0%). In addition, the proportion of children with impaired scores in reading comprehension despite having unimpaired skills in decoding, listening comprehension, and vocabulary was very small: 6 children (1.5% of the entire population).

Thirdly, there were strong differences between the groups of good and poor decoders for the prediction of reading comprehension. The effect of decoding skills on reading comprehension was significant in these two groups, but higher in poor decoders than in good decoders whereas the effect of phonemic awareness was significant only in poor decoders, not in good decoders. Alternatively, listening comprehension accounted for a significant part of the variance on reading comprehension only in good decoders, not in poor decoders. The results observed for the poor and good decoders are in agreement with hypotheses 3a, 3b and 3d as they indicate a greater effect on reading comprehension of both decoding skills and phonemic awareness than of listening comprehension in the group of poor decoders whereas the effect of the latter skill was greater than that of both decoding skills and phonemic awareness in the group of good decoders.

The results obtained by average decoders were close those of poor decoders for the effect of both decoding skills and phonemic awareness on reading comprehension. However, contrary to what was observed in the group of poor decoders, the effect of listening comprehension on reading comprehension was also high in that group, but less high than the one of decoding skills.

### Effect on reading comprehension of decoding skills versus listening comprehension in good, average and poor decoders

The results of the group of good decoders are consistent with those observed in a previous study on French-speaking first graders [11] in which, from the end of the first grade, the contribution of listening comprehension to the prediction of reading comprehension was greater than that of decoding skills. The opposite was observed in studies on English-speaking children [9–10], as well as with the entire group of the first graders used in the present study [1], and with two of the subgroups examined here: average decoders and, most importantly, poor decoders.

The difference in the relative weight of decoding versus listening comprehension on reading comprehension is generally attributed to orthographic transparency. Inconsistency is assumed to slow down reading development [7]; and the effect of orthographic consistency on such development can explain the greater contribution of decoding to reading comprehension in languages with a deep orthography, such as English [10].

As argued in a previous paper [1], the fact that the entire sample of the present study showed impairment in decoding skills, as did children from low SES families evaluated in other studies in English [37–38] and French [52] could explain why decoding was the most important predictor of reading comprehension in that study despite the relative transparency of French orthography [42–43]. The results of the French study suggesting that listening comprehension had greater contribution than decoding skills on reading comprehension at the end of the first grade [11] could be due to two factors that were not taken into account in this study: the children’s vocabulary level, and their high SES (the children enrolled in this study were from areas of Paris which are rather socially privileged). We can therefore assume that their environment allowed most of them to master decoding skills quickly and efficiently; this could have led to an increase in the weight of listening comprehension to the detriment of decoding skills in this study [11], as observed in the group of good decoders from the present study.

### Effect of phonemic awareness on reading comprehension in good, average and poor decoders

The unique contribution of phonemic awareness on reading comprehension was significant only in the groups of average and poor decoders. The results observed for these children are consistent with those of some studies conducted with young English-speaking children (e.g. [10] [23]).

Why in our study, conducted in a language with a shallower orthography than the English orthography, does phonemic awareness accuracy scores explain a unique proportion of the variance in reading comprehension, at least in the groups of average and poor decoders? We explain this result with the fact that, in the task used to assess reading comprehension, the context is not sufficient to infer the meaning of some words. In addition, to provide the correct answer it is often necessary to take into account subtle differences between closely related words at the level of grapheme-phoneme correspondences, such as those between the pronouns “le”, “la” and “les” (her/him/them). The finding that phonemic awareness accounted for a unique proportion of the variance in reading comprehension, but only for the children with average or poor decoding skills, makes sense in this context, in spite of the relative transparency of the French orthography.

### Effect of vocabulary on reading comprehension in good, average and poor decoders

As highlighted by some researchers [27], if what the child produces on a first attempt at reading a word does not sound like something that is in his/her oral vocabulary, then he/she has to change one or more GPCs and try again. This type of processing, which should be facilitated by a good level of vocabulary, should be mainly used at the beginning of learning to read (when word-level reading skills are not yet well developed), and would be higher in poor than in good decoders.

In the present study, although the interaction between grade level and vocabulary was non-significant, the contribution of vocabulary was only significant in poor and average decoders, but to a lesser extent in the former group than in the latter. These results are therefore partially consistent with our hypothesis 3c, as well as with some results of previous studies [27]. This makes sense if we assume that it is necessary to have reached a certain level of decoding skills in order to benefit from a lexical support.

### Prevalence of deficits in reading comprehension in good, average and poor decoders

The differences in the prevalence of a deficit in reading comprehension in children with good or average decoding skills compared to those with poor decoding skills indicate that decoding skills are strongly involved in reading comprehension, at least at the beginning of reading acquisition. In addition, only 6 children of the present cohort (1.53% of the entire population) have impaired skills in reading comprehension despite having unimpaired skills in decoding, listening comprehension and vocabulary.

The present results are consistent with those of another recent study with children from low SES families [22]. In this study, less than 1% of first- through third-grade students who scored poorly in reading comprehension had adequate skills in both decoding and vocabulary (listening comprehension was not assessed in this study). And, as stated by the authors “the term specific reading comprehension disability is a misnomer: Individuals with problems in reading comprehension that are not attributable to poor word recognition have comprehension problems that are general to language comprehension rather than specific to reading”. This is also what the results of the present study show.

### Conclusions

In conclusion, the present results support the fact that decoding skills play a crucial role in reading comprehension [4–5], and challenge the outcomes of studies on poor comprehenders [17–21] by showing that, at least in first grade, poor reading comprehension is very strongly linked to the level of decoding skills. They also show that it is possible to assess reading comprehension very early on in an orthography which is shallower than English (i.e., French), even in low SES children. Furthermore, the present results also highlight the fact that reading comprehension should be assessed at the same time as the three main predictors of that skill: decoding, listening comprehension and vocabulary. For virtually all children with reading comprehension difficulties, these assessments should help to understand the source of their difficulties in that domain, hence enabling the establishment of early specific remedial training. This is a crucial point: early training being the most effective [53–55].
